# Smartphone-Based Sensing System for Identifying Artificially Marbled Beef Using Texture and Color Analysis to Enhance Food Safety

**DOI:** 10.3390/s25144440

**Published:** 2025-07-16

**Authors:** Hong-Dar Lin, Yi-Ting Hsieh, Chou-Hsien Lin

**Affiliations:** 1 Department of Industrial Engineering and Management, Chaoyang University of Technology, Taichung 413310, Taiwan; s10715610@cyut.edu.tw; 2 Department of Civil, Architectural, and Environmental Engineering, The University of Texas at Austin, Austin, TX 78712-0273, USA; chslin@utexas.edu

**Keywords:** food safety, artificially marbled beef detection, smart sensing technology, human-centered healthcare, local binary patterns, texture analysis, color models, support vector machine

## Abstract

Beef fat injection technology, used to enhance the perceived quality of lower-grade meat, often results in artificially marbled beef that mimics the visual traits of Wagyu, characterized by dense fat distribution. This practice, driven by the high cost of Wagyu and the affordability of fat-injected beef, has led to the proliferation of mislabeled “Wagyu-grade” products sold at premium prices, posing potential food safety risks such as allergen exposure or consumption of unverified additives, which can adversely affect consumer health. Addressing this, this study introduces a smart sensing system integrated with handheld mobile devices, enabling consumers to capture beef images during purchase for real-time health-focused assessment. The system analyzes surface texture and color, transmitting data to a server for classification to determine if the beef is artificially marbled, thus supporting informed dietary choices and reducing health risks. Images are processed by applying a region of interest (ROI) mask to remove background noise, followed by partitioning into grid blocks. Local binary pattern (LBP) texture features and RGB color features are extracted from these blocks to characterize surface properties of three beef types (Wagyu, regular, and fat-injected). A support vector machine (SVM) model classifies the blocks, with the final image classification determined via majority voting. Experimental results reveal that the system achieves a recall rate of 95.00% for fat-injected beef, a misjudgment rate of 1.67% for non-fat-injected beef, a correct classification rate (CR) of 93.89%, and an F1-score of 95.80%, demonstrating its potential as a human-centered healthcare tool for ensuring food safety and transparency.

## 1. Introduction

The growing prevalence of food fraud, particularly the use of fat injection to mimic the marbling of high-quality Wagyu beef, raises serious concerns about consumer health and trust in the food supply. Mislabeled products may contain unverified additives or allergens, posing potential health risks. Aligned with the principles of human-centered healthcare, which emphasize informed decision making, this study presents a smartphone-based smart sensing system for detecting artificially marbled beef. By enabling consumers to capture and classify beef images in real time using texture and color analysis with a machine learning classifier, the system supports food safety and dietary transparency through accessible, consumer-facing technology [1,2].

Japanese Wagyu beef is renowned for its rich fat content, uniform marbling, and tender texture, commanding premium prices in the market. To capitalize on this demand, lower-cost artificially injected beef—marketed as “Wagyu-grade”—has emerged, mimicking the visual characteristics of authentic Wagyu. Many vendors misrepresent such products to gain higher profits, often without regulatory consequences, as the labeling of processed meats is not universally required. This deception leads consumers to unknowingly purchase lower-quality, processed beef at premium prices [3].

Injected beef is a processed meat product created by injecting melted beef fat—often with flavor-enhancing additives—into lean cuts to improve texture, taste, and market value [4]. Originating in Japan, this technique allows low-cost beef to resemble premium cuts but also increases the risk of microbial contamination, necessitating frozen storage and thorough cooking [5]. While the method offers consumers a more affordable alternative to high-grade beef, it is vulnerable to misuse by vendors who deceptively market it as premium meat, misleading consumers and inflating prices.

### 1.1. Appearance Difference Between Japanese Wagyu Beef and Fat-Injected Beef

Beef fat injection technology was originally developed to enhance the quality of lower-grade meat but has since been misused to imitate the appearance of high-grade Wagyu, leading to mislabeling and consumer deception. While both Wagyu and fat-injected beef exhibit dense marbling, Wagyu typically shows branched, scattered fat flecks, whereas injected beef presents linear, uniform, and interconnected fat patterns [6,7,8]. As shown in Table 1, these visual differences can assist in identification. To address this issue, the present study introduces a similarity detection system that analyzes marbling features to help consumers verify beef authenticity and prevent food fraud.

### 1.2. Research Motivation and Purpose

The widespread mislabeling of fat-injected beef as premium Wagyu has heightened food safety concerns and fraud risks, potentially exposing consumers to hidden additives and allergens. This study proposes a smartphone-based sensing system that integrates texture and color analysis with machine learning to detect fat-injected beef in real time. It hypothesizes that combining these features will enable accurate differentiation of fat-injected beef from naturally marbled beef and that the selected classifier will outperform others due to its effectiveness with small datasets. The goal is to develop and validate this system to enhance food transparency and support consumer protection.

### 1.3. Study Limitations

This study’s ability to differentiate Wagyu from fat-injected beef is limited by several practical factors. The system’s accuracy is affected by variations in beef cuts, packaging methods, and imaging conditions such as angle and size. Its applicability is further restricted to uncooked beef, as cooking alters lean meat color and melts fat, making visual distinction impossible post heating. Packaging-related issues such as glare or reflections and background interference from labels or obstructions can reduce detection precision and require user effort to ensure image quality. These limitations underscore the need for improved preprocessing techniques and user guidance to enhance system reliability and support food safety in consumer-centered applications.

This paper first reviews existing methods for beef marbling detection, then introduces the proposed machine learning models based on texture and color features for identifying artificially marbled beef. It proceeds with performance evaluations and robustness analysis, followed by a summary of key contributions and suggestions for future research.

## 2. Literature Review

This literature review explores current methods for evaluating beef quality, including marbling, freshness, tenderness, and fat distribution. While techniques such as histological, chemical, and imaging analyses are widely used, few address the detection of artificially marbled beef. This gap highlights the need for smart sensing solutions, such as smartphone-based systems, to improve transparency and protect public health.

### 2.1. Overview of Beef Inspection and Quality Grades

Beef is a nutritionally rich and high-priced meat, making it susceptible to fraud through processing and resale at premium prices. As fat content varies by breed and cut, key quality indicators include tenderness, juiciness, and marbling distribution, driving the development of diverse inspection methods and standards.

Hosseinpour et al. [9] developed an image processing system with rotation, translation, and scale invariance to extract texture features under varying lighting, using an ANN to predict beef tenderness. ElMasry et al. [10] and Velásquez et al. [11] applied hyperspectral imaging for rapid beef classification and marbling analysis. Jackman et al. [12] used texture features from color components to assess palatability based on marbling and texture. Chen et al. [13] introduced a visual thresholding method to separate fat in ribeye images. Arsalane et al. [14] proposed a portable freshness assessment tool using hue, chroma, and saturation data with PCA and SVM. Cheng et al. [15] reviewed marbling detection methods since 2000, highlighting a shift from visual and chemical techniques to emerging spectral imaging approaches.

Computer vision has been widely used in beef inspection for tasks such as assessing freshness [16,17], tenderness, fat distribution [18], palatability [19], and quality grading [20,21]. However, few studies address quality inspection of raw beef after human processing, such as artificial marbling—a practice that raises food safety concerns and undermines consumer trust. Often mislabeled as premium Wagyu, artificially marbled beef presents a key challenge this study seeks to overcome using smart sensing technology.

### 2.2. Detection Techniques for Beef Marbling

Detecting artificial marbling in beef at laboratory or industrial levels requires accurate, objective methods to differentiate injected fat from natural marbling [15,22]. This study reviews physical, chemical, and imaging-based techniques used in scientific and industrial settings, highlighting their suitability for identifying artificial enhancements across various operational contexts.

Histological analysis examines stained beef tissue microscopically to assess fat distribution, offering accuracy but lacking practicality for real-time grading due to its time-consuming process [23,24]. Similarly, chemical composition analysis accurately detects anomalies in lipid content and fatty acid profiles but is costly and time-intensive, making it more suitable for regulatory or fraud detection purposes [25,26].

Imaging-based methods offer advanced, non-destructive alternatives. Hyperspectral imaging (HSI) maps spatial and chemical properties for rapid inline grading but involves high costs, complex calibration, and intensive data processing [11,27]. Structured illumination reflectance imaging (SIRI) enhances subsurface texture detection to distinguish injected fat from natural marbling, though it requires specialized equipment, is environment-sensitive, and has limited depth penetration [28,29].

X-ray micro-CT provides detailed 3D imaging of fat distribution, useful for research or premium quality checks, but its high cost, bulkiness, and radiation risks limit high-throughput use [30,31]. Near-Infrared Spectroscopy (NIRS) offers a portable, fast method for spot-checking fat and muscle content, though it requires frequent calibration, has shallow penetration, and is sensitive to moisture [32,33].

For industrial use, HSI and NIRS are favored for their speed and non-destructive analysis, while histological and chemical methods serve as accurate, lab-based tools. Together, these methods offer a balanced toolkit for detecting artificial marbling. However, limitations in imaging approaches underscore the need for advancements to enhance practicality across industrial, laboratory, and consumer applications.

### 2.3. Texture-Based Analysis Methods for Beef Marbling

Texture analysis is essential for evaluating beef marbling, a key factor affecting tenderness, juiciness, and flavor. Local binary pattern (LBP) is widely used for its simplicity, efficiency, and ability to capture fine texture details [34]. Variants like uniform, rotation-invariant, and multi-scale LBP enhance robustness to noise and image variations [35]. Other methods such as gray level co-occurrence matrix (GLCM) [36], Gabor filters [37], and wavelet transforms [38] extract statistical and directional texture features. These techniques are often combined with color features (e.g., RGB, HSV, and Lab*) to better distinguish natural marbling from artificial fat injection patterns [39].

Recent advances in deep learning have introduced convolutional neural networks (CNNs) for automated marbling assessment, enabling the extraction of complex texture patterns without manual feature design and achieving high accuracy [28]. Hybrid models combining traditional features like LBP with CNNs further enhance performance under varied conditions [40]. While CNNs offer superior results, they require large labeled datasets and high computational power. The most effective systems integrate texture and color features, block-based analysis, and robust classifiers such as SVM or random forest, supporting accurate and scalable marbling evaluation for both industrial and consumer use.

### 2.4. Color-Based Analysis Models for Beef Marbling

Color analysis plays a key role in automated beef marbling evaluation by highlighting the visual contrast between intramuscular fat (IMF) and lean muscle. The RGB color space is widely used due to its compatibility with digital imaging but is sensitive to lighting variations [41,42]. The HSV model improves robustness by separating color and brightness, effectively distinguishing fat from muscle [43]. The CIE Lab* space, known for perceptual uniformity, aligns well with human vision, with L* indicating lightness and a*/b* capturing color shifts [44]. These color features are typically quantified using statistical metrics such as mean, standard deviation, histograms, and color moments to characterize marbling.

Color features are commonly used in marbling classification with machine learning models such as SVM, random forest, and logistic regression. Combining color and texture features (e.g., LBP + RGB, GLCM + L*a*b*, etc.) improves accuracy, especially in distinguishing natural from injected marbling. Liu et al. [45] achieved high accuracy using RGB features in a CNN, while Olaniyi et al. [46] effectively identified injected fat with HSV and L*a*b* inputs. Smartphone-based systems often use RGB data with preprocessing to adjust for lighting. Public datasets from Hosseinpour et al. [9] and Lin et al. [47] support reproducibility. Integrating color features into deep learning remains a promising path for real-time, consumer-grade marbling detection.

### 2.5. Machine Learning Approaches for Classification

Machine learning offers an objective, consistent approach to beef marbling classification, complementing or replacing traditional human grading. Models like SVM, Random Forest (RF), and k-Nearest Neighbors (k-NN) are widely used for structured features derived from color, texture, or shape. SVMs perform well in high-dimensional spaces and are commonly paired with features like LBP and RGB histograms, as shown by Chen et al. [41] in predicting beef fat color. Random Forests provide robust ensemble learning, effectively handling heterogeneous data and avoiding overfitting, as demonstrated in beef freshness studies using hyperspectral and physicochemical features [48]. These models are favored for their balance of accuracy, interpretability, and efficiency on moderate-sized datasets.

With the rise of annotated beef image datasets, deep learning, particularly CNNs, has become central to marbling classification by automatically learning features from raw images. Liu et al. [45] applied an enhanced YOLOv8x model, while hybrid methods like that of Tong and Tanaka [49] combine deep and traditional models to improve performance on smaller datasets. Transfer learning with pre-trained networks like ResNet and MobileNet enable efficient adaptation for marbling tasks, supporting mobile and embedded use [9,47]. As real-time grading and fraud detection gain importance, integrating lightweight models, edge computing, and domain-specific learning will be key for practical deployment in both industrial and consumer contexts.

The literature highlights food safety as a global concern [1,2], underscoring the importance of food inspection. While existing studies focus on marbling detection [11,15,18], freshness [14], tenderness [10,16,17], and grading [20,21], none address whether beef fat has been artificially processed—an issue this study aims to investigate.

The constraints of imaging-based methods underscore the critical need for technological advancements to enhance their practicality, particularly for consumer use, which is vital for ensuring food safety. Enabling consumers to detect artificial marbling in beef through accessible tools like smartphone-based systems empowers them to make informed purchasing decisions, reducing the risk of exposure to mislabeled products that may contain unverified additives or allergens, thereby directly safeguarding public health and promoting transparency in the food supply chain.

## 3. Proposed Methods

**Figure 1:**
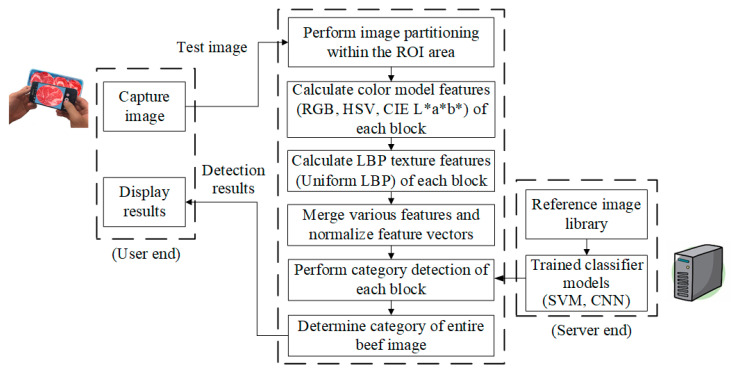
The overall system concept diagram of this study.

This study proposes a smartphone-based system for detecting artificially marbled beef by integrating mobile imaging with cloud-based processing. As illustrated in Figure 1, the system workflow begins with the user capturing an image, which is transmitted to a central server for analysis. The image is segmented within a predefined region of interest (ROI), and both color model and LBP texture features are extracted from each grid block. These features are combined into a comprehensive vector for block-level classification using a trained machine learning model. The aggregated results determine the overall beef category, referencing a server-side image library. Final classification outcomes are returned to the user in real time, supporting informed purchasing decisions. The system continuously evaluates performance metrics to refine parameters and optimize detection accuracy.

### 3.1. ROI Extraction and Gridding in a Beef Image

**Figure 2:**
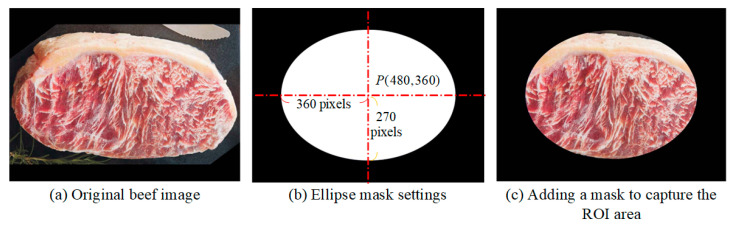
Adding a mask to the captured image and extracting the ROI area.

To minimize variability in image capture across users, this study applies a black elliptical mask during acquisition, as shown in Figure 2. Images are assumed to have a resolution of 960 × 720 pixels and may include background elements. The mask, centered at P(480, 360), spans 75% of the image dimensions, with a major axis of 720 pixels along the X-axis and a minor axis of 540 pixels along the Y-axis, as illustrated in Figure 2b. The masked image, shown in Figure 2c, defines the region used for subsequent processing.

**Figure 3:**
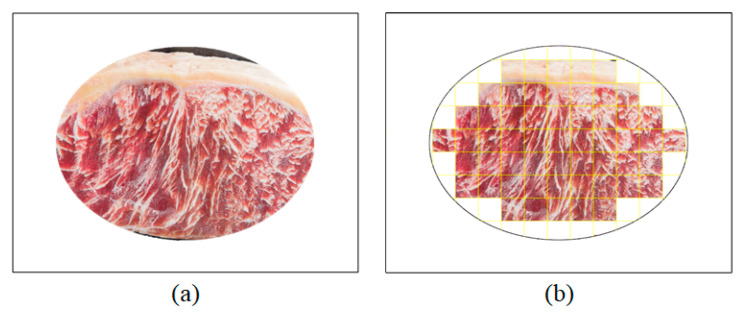
Dividing the ROI image into grids: (**a**) a ROI image; (**b**) a block image with whole grids fully containing beef after dividing the ROI image.

After applying the mask to extract the ROI, the image is divided into grid blocks denoted as b_i_(x, y), which serve as the basic units for feature extraction and classification. The block size significantly affects performance; blocks that are too small fail to capture sufficient texture, while overly large blocks reduce local detail. This study adopts an initial block size of 64 × 64 pixels with a 4-pixel interval on each side to evaluate the impact of block size on classification effectiveness. As illustrated in Figure 3, each ROI yields 55 valid grid blocks excluding masked areas, and these b_i_(x, y) units form the foundation for subsequent analysis.

### 3.2. Feature Extraction in a Gridded ROI Beef Image

After dividing the ROI into grid blocks, this study extracts both texture and color features from each block. Color features are derived from RGB, HSV, and CIE L*a*b* color spaces, while texture features are captured using Uniform LBP. These combined features form the basis for classifying beef images.

#### 3.2.1. Color Models of Beef Color Features

**Figure 4:**
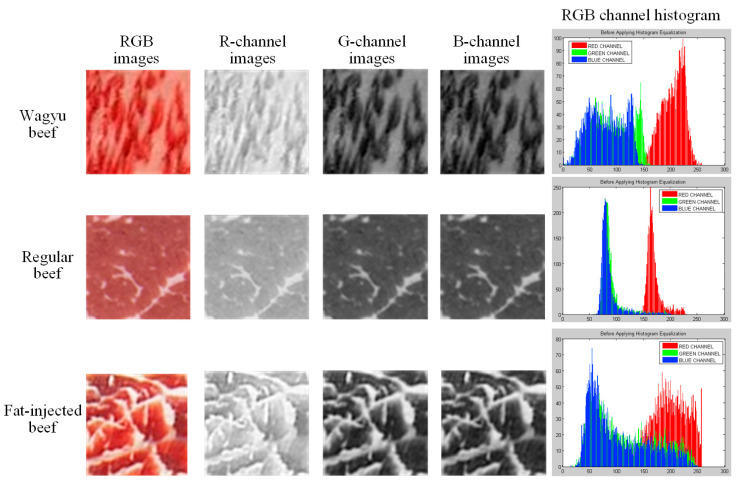
RGB image, each channel image, and RGB channel histogram of the grid block.

RGB is a widely used color space composed of red, green, and blue channels, which combine to form color images. In this study, each grid block image b(x,y) is analyzed by extracting its RGB components. To capture color differences among beef types, the mean and standard deviation of each RGB channel are calculated for every block. As shown in Figure 4, the red channel exhibits significantly higher brightness, while the green and blue channels show high overlap with minimal variation. These statistical features are used to distinguish between different beef categories.

**Figure 5:**
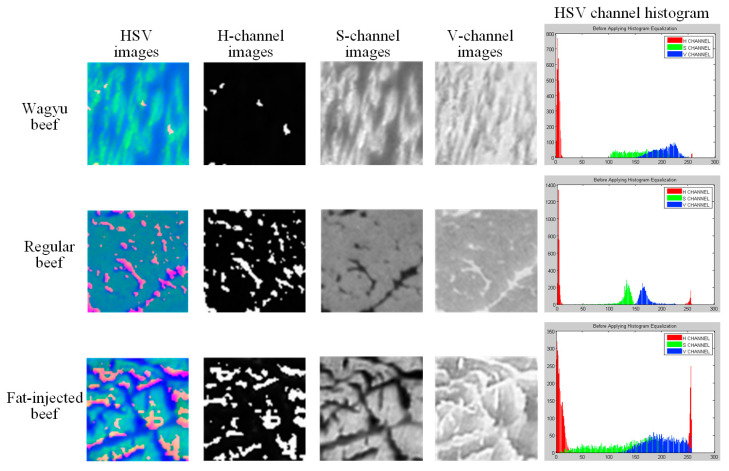
HSV image, each channel image, and HSV channel histogram of the grid block.

HSV is a color model that represents RGB values in a conical coordinate system, where H (hue) indicates color type (0–360°), S (saturation) represents color purity (0–100%), and V (value) reflects brightness (0–100%). In this study, RGB images are converted to HSV, and each component is normalized to a 0–255 scale. As shown in Figure 5, the H, S, and V components are visualized separately with corresponding histograms. The H component shows a wide distribution and high standard deviation, while the V component has a higher mean than S. Visually, the H component appears black-and-white with the highest contrast, though it does not effectively distinguish fat from lean meat in Wagyu beef. The V component offers a clearer contrast than S, making it more suitable for identifying marbling patterns.

**Figure 6:**
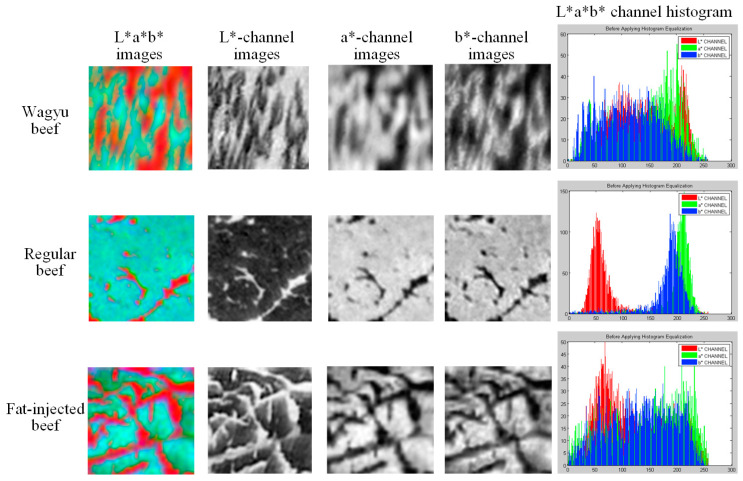
L*a*b* image, each channel image, and L*a*b* channel histogram of the grid block.

The CIE L*a*b* color space models human visual perception more accurately than RGB, making it suitable for color analysis and adjustment. In this model, L* represents brightness, a* spans the red–green axis, and b* spans the blue–yellow axis. This study converts beef images from RGB to CIE L*a*b* and normalizes each component to a 0–255 scale. As shown in Figure 6, the L*, a*, and b* component images and their histograms are presented. For Wagyu and fat-injected beef, the histograms show even distributions and high overlap across all components. In contrast, general beef exhibits a lower L* mean and smaller standard deviation differences among the components, suggesting less brightness variation and reduced visual contrast.

**Figure 7:**
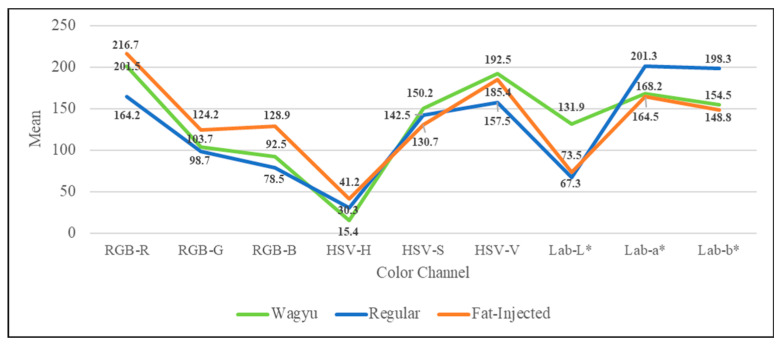
Mean value plot for each color channel (RGB, HSV, and CIE L*a*b*) across the three beef categories (Wagyu, regular, fat-injected) derived from the 240-image dataset.

**Figure 8:**
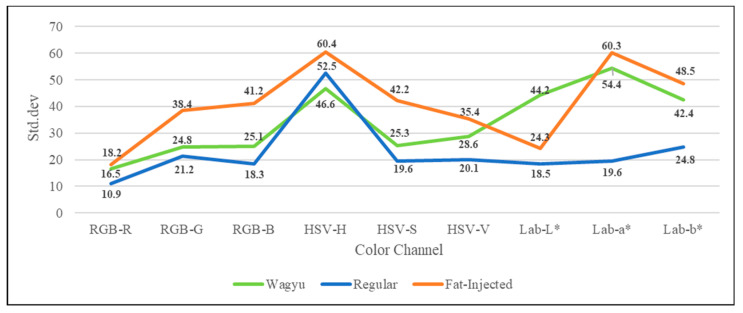
Standard deviation plot for each color channel (RGB, HSV, and CIE L*a*b*) across the three beef categories (Wagyu, regular, fat-injected) derived from the 240-image dataset.

This study extracts component images from experimental samples using RGB, HSV, and CIE L*a*b* color modes and calculates corresponding statistical measures. Figure 7 and Figure 8 show the plots of means and standard deviations for each color channel (RGB, HSV, and CIE L*a*b*) across the three beef categories (Wagyu, regular, and fat-injected) derived from the 240-image dataset (80 per category), respectively. In the RGB model, all beef types share higher R component values compared to G and B, with G and B histograms showing high overlap, but regular beef exhibits lower standard deviations across components than Wagyu and fat-injected beef. For the HSV model, similarities include H component values concentrating at the extremes and a higher V component mean over S, while fat-injected beef displays greater variability in all components compared to Wagyu and regular beef. In the Lab* model, Wagyu and fat-injected beef share widely spread histograms with high variability, whereas general beef shows low variability, with its L* component mean being lower than a* and b*. These findings highlight the distinct color characteristics of beef types, supporting the study’s color analysis approach for detecting artificially marbled beef.

#### 3.2.2. LBP Model of Beef Texture Features

LBP is a texture descriptor known for its rotation and illumination invariance and low computational cost [34]. This study employs uniform pattern LBP to extract texture features from 64 × 64 pixel grid blocks within the ROI. The RGB images are first converted to grayscale to simplify processing and then divided into 55 blocks per image. Only blocks fully contained within the elliptical mask are used, ensuring that feature extraction focuses exclusively on beef texture.

LBP quantifies the texture of an image based on the intensity relationships between a central pixel and its neighbors. The LBP computation is performed for each grid block. This involves comparing the intensity of each pixel with *P* neighboring pixels at radius *R* (e.g., *P* = 8; *R* = 1 for its neighbors in a 3 × 3 neighborhood), typically using eight neighbors. A binary value is assigned as −1 if the neighbor’s intensity is greater than or equal to the center pixel and 0 otherwise, forming a binary pattern arranged in a circular order (clockwise or counterclockwise). This pattern is then converted into a decimal value, yielding an LBP code ranging from 0 to 2*^P^* − 1 (e.g., 0 to 255 for eight neighbors). The LBP code LBP*_P,R_* encodes local texture by comparing pixel intensities, with 2*^P^* weighting each bit position.

Uniform LBP refines this process by focusing on patterns with limited transitions, reducing dimensionality while preserving robustness to rotation and illumination changes. The study employs the uniform LBP variant, which identifies patterns with at most two transitions between 0 and 1 (e.g., 00000000, 11111111, 00001111, etc.), grouping non-uniform patterns into a single bin. This reduces the feature space from 256 to 59 (58 uniform patterns plus 1 non-uniform bin), addressing the issue of excessive pattern types. The transition count *U*(LBP) ensures only patterns with up to two changes are considered uniform, reducing noise sensitivity.

**Figure 9:**
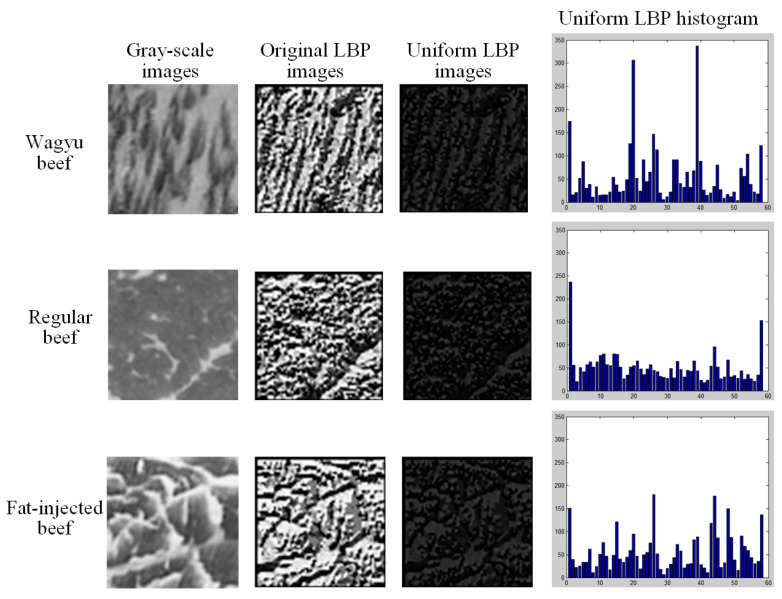
Grayscale image, original LBP and uniform LBP images, and uniform LBP channel histogram of the grid block.

After computing the LBP for each grid block, a 59-bin histogram is generated to represent texture features, with 58 bins for uniform patterns and 1 for non-uniform patterns. Each pixel is replaced by its LBP code, and the resulting histogram is normalized by the total pixel count to ensure scale invariance. This 59-dimensional vector serves as the uniform LBP feature for the grid block, providing a compact and robust representation of its texture. Figure 9 displays grayscale grid block images from different beef categories, comparing original LBP and uniform LBP representations. Uniform LBP reduces the number of binary pattern types derived from the original LBP, simplifying texture representation. As a result, uniform LBP images appear with reduced brightness and contrast compared to original LBP images and are ultimately converted into histograms for analysis.

The feature extraction process is applied to all 55 grid blocks within each ROI image, generating 59-dimensional uniform LBP histograms per block. These are either used individually or concatenated into a single 3245-dimensional (55 × 59) feature vector to capture comprehensive texture information. The uniform LBP features are then combined with color features (e.g., RGB) to form a unified input for the classification system, enabling accurate differentiation among beef categories such as Wagyu, fat-injected, and general beef.

### 3.3. Machine Learning Models Applied to Artificially Marbled Beef Detection

This study employs three color models and uniform LBP texture features to characterize beef images and evaluates various feature combinations using a small-sample dataset. The combinations are listed in Table 2, with corresponding experimental results presented in the following section.

#### 3.3.1. SVM Model

This study combines LBP texture and color features for classifying beef into three categories using a support vector machine (SVM). To prevent large numerical differences among features from affecting classification, all extracted feature vectors are normalized prior to input. Normalization enhances SVM performance by ensuring consistency across feature dimensions. In this study, feature values are scaled to the [0, 1] range to improve classification stability and mitigate the impact of varying units.

**Figure 10:**
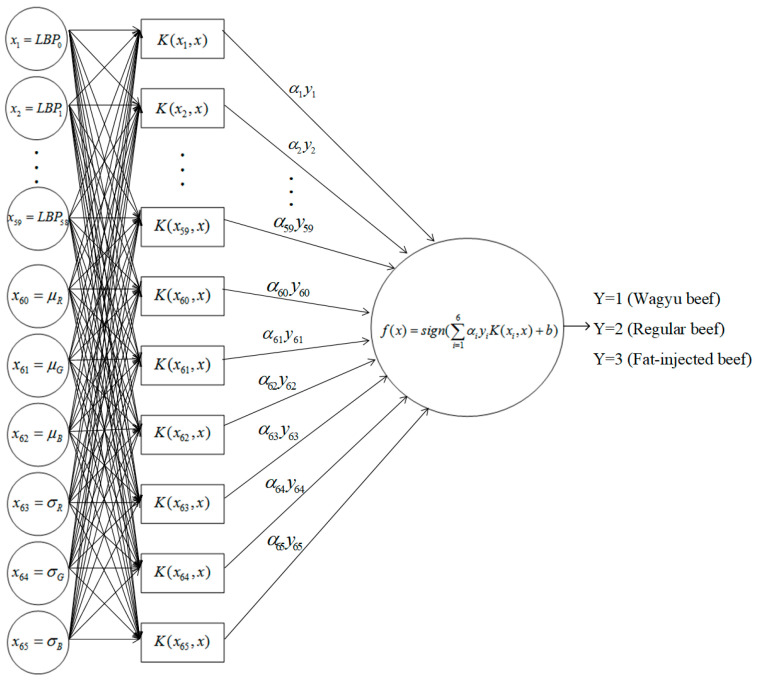
Schematic diagram of the SVM model in this study.

This study develops a beef classification system using an SVM model. Normalized feature vectors are input into the SVM for training, as shown in Figure 10. The system processes the combined features from each grid block to detect artificially marbled beef. The SVM uses an RBF kernel, with performance optimized by tuning the penalty coefficient *C* and kernel coefficient *γ*. The optimal parameter values for identifying artificially marbled beef are determined, with the parameter setting combinations presented in Table 3.

In the radial basis function (RBF), the parameter *C* adjusts the confidence interval range during the network’s learning process, while the parameter *r* determines the distribution of data after mapping to a new feature space within the RBF kernel function. Thus, parameters *C* and *r* are the two most critical factors in the radial basis function. This study explores various combinations of these two parameters to identify the optimal parameter settings for the SVM network. The RBF kernel function is presented in Equation (1), where *α*_i_ represents the Lagrange multiplier corresponding to the support vector x_i_, y_i_ denotes the class label (1, 2, or 3) of the support vector x_i_, *b* indicates the output bias, and *g*(*x*) represents the beef grid block in this study. Equation (3) defines the RBF kernel function *K*(x_i_, x_j_) used in Equation (1).

(1) $$f(x)=sign\left(\sum_{i=1}^{65}\alpha_{i}y_{i}K(x_{i},x_{j})+b\right)$$

(2) $$g(x)=\left\{\begin{array}{lll} 1, & if & f(x)=0\\ 2, & if & f(x)>0\\ 3, & if & f(x)<0 \end{array}\right.$$

(3) $$K(x_{i},x_{j})=K\left(\left(\mu_{LBP_{0_{i}}}\right),\left(\mu_{LBP_{0_{j}}}\right)\right)=\exp\left(-\gamma {\left\|\mu_{LBP_{0_{i}}}-\mu_{LBP_{0_{j}}}\right\|}^{2}\right)$$

To determine the optimal combination of the two parameter values [*C*, *r*] in the radial basis function for this study, experiments were conducted with the parameter domain [2^−8^, 2^8^], and the performance results for each parameter value combination are detailed in the experiments and results section.

#### 3.3.2. CNN Model

With the rise of deep learning, convolutional neural networks (CNNs) have become a mainstream classification approach, eliminating the need for manual feature extraction by automatically learning features from input images. However, CNNs require large datasets to achieve high accuracy. To compare performance, this study also applies a CNN model to the same beef image samples used in previous classifier experiments.

**Figure 11:**
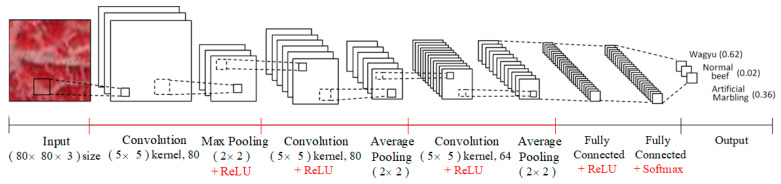
Test procedure of the CNN model for beef classification.

Figure 11 shows the architecture of the CNN model used in this study. Divided beef sample images serve as inputs, processed through three convolutional layers with 5 × 5 kernels, each followed by pooling to reduce dimensionality while preserving key features. The first pooling layer uses max pooling to retain texture details, while the second and third use average pooling to reduce variance and preserve background information. ReLU activation functions are applied between layers to introduce non-linearity and prevent vanishing gradients. Finally, a fully connected layer transforms the extracted features into a one-dimensional vector, with a Softmax function computing the classification probabilities for each beef category. For the CNN model in this study, the primary adjustable parameter is the block size used during image segmentation. Other CNN parameters, such as network architecture and learning settings, are held constant during performance comparison. Unlike SVM, which relies on extracted LBP and color features, CNN processes raw pixel information directly from image blocks.

### 3.4. Artificially Marbled Beef Detection System

**Figure 12:**
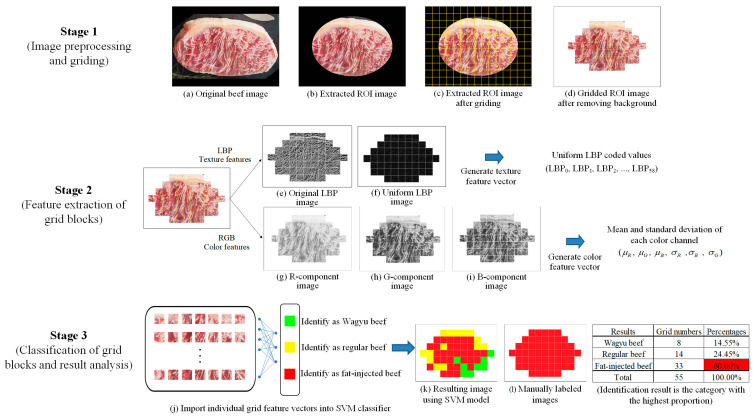
Stage diagram of an artificially marbled beef detection system.

This study develops a technology-based system to help consumers identify beef categories and detect artificially marbled beef. Images are captured under varying lighting and shooting conditions, with the target beef region confined within a predefined mask. The masked area is divided into grid blocks, each undergoing feature extraction and classification. Final results are determined through majority voting based on block-level predictions. The complete workflow is illustrated in Figure 12.

#### 3.4.1. Image Capture and System Requirements

**Figure 13:**
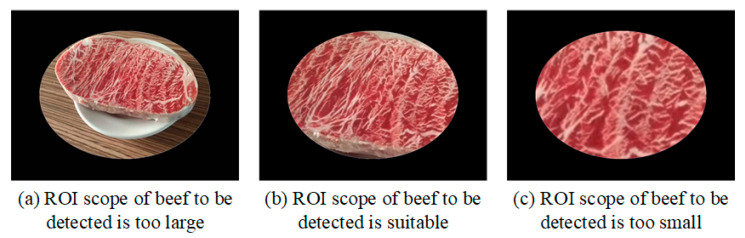
Examples of appropriate and inappropriate imaging ranges for test images.

This study aims to support future integration with a mobile app for image capture and recognition by using sample images taken under various lighting and environmental conditions. Masks are applied to simulate handheld image capture. To reduce variability from uncontrolled capture conditions, selected images ensure that the detection region is clearly visible within the defined ROI mask and appropriately sized. Figure 13 illustrates examples: (a) an overly large ROI including background noise, (b) a properly sized ROI, and (c) an overly small ROI that may hinder effective feature extraction.

This study uses 240 images for small-sample experiments to determine system parameters and 600 images for large-sample experiments to assess performance. The detection system is developed using MATLAB R2019b on a Laptop with CPU: AMD Ryzen™ 9 5900HS, 48 GB RAM; GPU: GeForce^®^ RTX 3060; and operating system: Windows 10. It segments ROI images into grid blocks, extracts features, and classifies each block using an SVM model. Final identification results are evaluated using performance metrics.

#### 3.4.2. Performance Evaluation Metrics

To evaluate the classification performance of the proposed beef detection system, this study uses Type I error (α), Type II error (β), and correct classification rate (CR), along with precision, recall, and the F1-score—the harmonic mean of precision and recall. A higher F1-score indicates better overall detection performance. Evaluation is conducted in two stages: (1) block-level, assessing individual grid block classification, and (2) image-level, assessing overall image classification.

1. Performance evaluation based on the block level

The misjudgment rate (b_α)% for non-fat-injected beef is defined as

(4) $$Misjudgment\;rate\;(b\_\alpha )\%=\left(\frac{Number\;of\;misjudged\;blocks\;among\;blocks\;detected\;as\;fat-injected\;beef}{Total\;number\;of\;blocks\;for\;real\;wagyu\;beef\;and\;regular\;beef}\right)\times 100\%$$

The detection rate (b_1 − β)% or recall (b_R)% for fat-injected beef is defined as

(5) $$Detection\;rate\;(b\_1-\beta )\%=\left(\frac{Number\;of\;blocks\;correctly\;classified\;as\;fat-injected\;beef}{Total\;number\;of\;blocks\;for\;real\;fat-injected\;beef}\right)\times 100\%$$

Precision (b_P)% for fat-injected beef is defined as

(6) $$Precision\;(b\_P)\%=\left(\frac{Number\;of\;blocks\;correctly\;classified\;as\;fat-injected\;beef}{Total\;number\;of\;blocks\;detected\;as\;fat-injected\;beef}\right)\times 100\%$$

The classification rate (b_CR%) for all grid blocks in test images is defined as

(7) $$Classification\;rate\;(b\_CR\%)=\left(\frac{Number\;of\;correctly\;classified\;grid\;blocks}{Total\;number\;of\;blocks\;for\;test\;images}\right)\times 100\%$$

2. Performance evaluation based on the image level

The misjudgment rate (α)% for non-fat-injected beef is defined as

(8) $$Misjudgment\;rate\;(\alpha )\%=\left(\frac{Number\;of\;misjudged\;images\;among\;images\;detected\;as\;fat-injected\;beef\;images}{Total\;number\;of\;images\;for\;real\;Wagyu\;beef\;and\;regular\;beef}\right)\times 100\%$$

The detection rate (1 − β)% or recall (R)% for fat-injected beef is defined as

(9) $$Detection\;rate\;(1-\beta )\%=\left(\frac{Number\;of\;images\;correctly\;classified\;as\;fat-injected\;beef}{Total\;number\;of\;images\;for\;real\;fat-injected\;beef}\right)\times 100\%$$

Precision (P)% for fat-injected beef is defined as

(10) $$Precision\;(P)\%=\left(\frac{Number\;of\;images\;correctly\;classified\;as\;fat-injected\;beef}{Total\;number\;of\;images\;detected\;as\;fat-injected\;beef}\right)\times 100\%$$

The classification rate (CR)% for all test images is defined as

(11) $$Classification\;rate\;(CR)\%=\left(\frac{Number\;of\;correctly\;classified\;images}{Total\;number\;of\;test\;images}\right)\times 100\%$$

F1-score (F1-Score)% for fat-injected beef based on the number of grid blocks or images is defined as

(12) $$F1\;Score\;(F1-Score)\%=\left(\frac{2\times Recall\times Precision}{Recall+Precision}\right)\times 100\%$$

The F1-score is calculated specifically for the fat-injected beef category in a one-vs-rest framework, treating Wagyu and regular beef as a single non-fat-injected class, to emphasize the system’s performance in detecting artificial marbling.

Since image-level performance is determined by majority voting across segmented blocks, ties may occur when two or more categories have the same number of blocks. To resolve this, the system applies a priority rule: fat-injected beef is classified first, followed by regular beef, then Wagyu beef. This ensures a stricter and more conservative classification approach.

## 4. Experiments and Results

To validate the proposed method for detecting artificially marbled beef and classifying beef categories, this study conducts practical experiments and performance evaluations. Results are compared with other methods to assess effectiveness, followed by a sensitivity analysis to examine additional influencing factors.

### 4.1. Parameter Optimization Results

The proposed method requires parameter optimization for components such as block size and LBP operator settings. To identify optimal values, preliminary experiments were conducted using 240 images (960 × 720 pixels each), with each image divided into 36 grid blocks. Table 4 shows the quantity distribution of sample images and grid blocks used in the experiments.

#### 4.1.1. Parameter Setting of Grid Block Size

**Figure 14:**
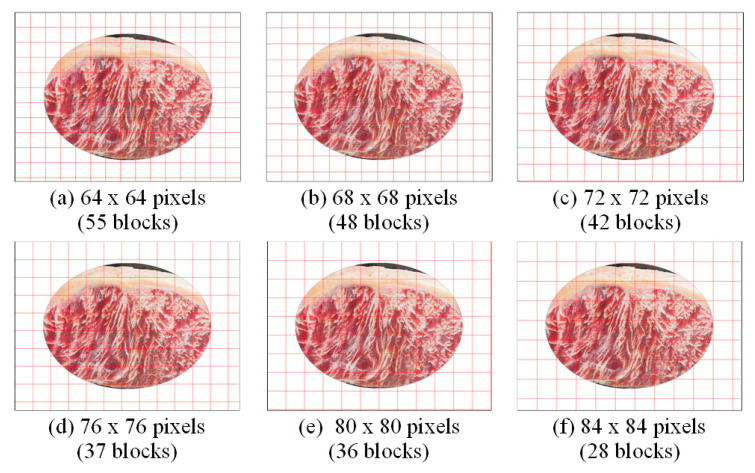
ROI images with different grid block sizes.

To extract local features from beef images, this study divides ROI-masked images into non-overlapping, equally sized blocks, which serve as the basic units for analysis. Block size significantly influences feature extraction and classification accuracy: blocks that are too small may miss overall texture patterns, while overly large blocks may obscure local detail. To identify an optimal size, the study begins with a block size of 64 × 64 pixels and increases in 4-pixel increments on the sides to evaluate performance impacts. Figure 14 shows the partitioning results and the number of complete blocks per image for each block size.

**Figure 15:**
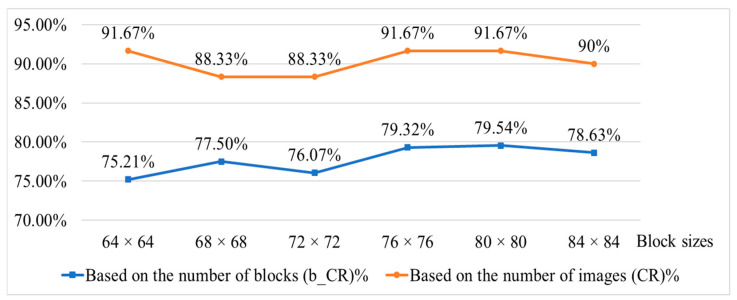
Comparison of the classification rates using different block sizes based on block level and image level.

Table 5 compares performance across different block sizes using both block-based and image-based approaches. Results show that a block size of 80 × 80 pixels achieves a lower misjudgment rate for non-injected beef ((b_α)% and (α)%) and a higher detection rate for fat-injected beef ((b_1 − β)% and (1 − β)%). Figure 15 further supports the above findings using the classification rate indicator. Therefore, based on this analysis, 80 × 80 pixels is selected as the optimal block size for the proposed system.

#### 4.1.2. Parameter Setting of LBP Feature Operator

To address texture variations at different scales and frequencies in beef images, this study evaluated three LBP operator configurations: LBP(8, 2), LBP(8, 1), and LBP(16, 2), where P is the number of sampling points, and R is the sampling radius. As shown in Table 6, LBP(8, 1) achieved the highest block-based detection rate for fat-injected beef (b_1 − β)% and the best block-based classification rate (b_CR)%. Thus, LBP(8, 1) is selected as the optimal operator for texture feature extraction in this study.

#### 4.1.3. Parameter Setting of SVM Classification Model

**Figure 16:**
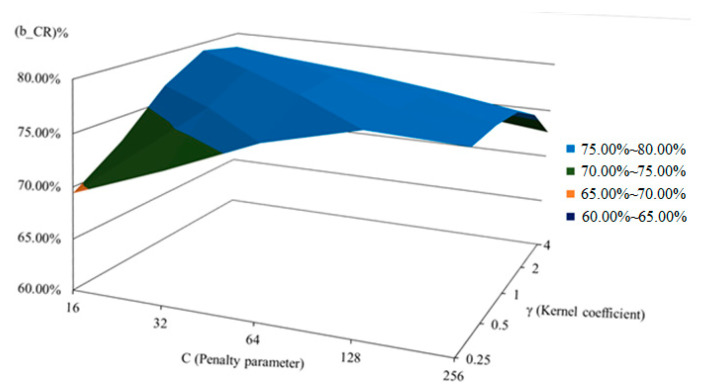
Surface plot of classification rates based on block level using different parameter combinations of the SVM classification model.

This study employs an SVM with a radial basis function (RBF) kernel for image classification. The key parameters, penalty parameter (*C*) and kernel coefficient (*γ*), are optimized through a grid search over the range [2^−8^, 2^8^]. The best results are achieved with *C* between 2^4^ and 2^8^ and *γ* between 2^−2^ and 2^2^. As shown in Table 7 and Figure 16, the optimal configuration is (*C*, *γ*) = (64, 1), which is adopted for the proposed system. 

#### 4.1.4. Feature Vector Setting for Different Feature Pattern Combinations

In this study, feature extraction is applied to divide grid blocks prior to classification, using two feature types, texture and color. Color features are evaluated across different color spaces (RGB, HSV, and CIE L*a*b*), each offering unique descriptive capabilities. To assess system performance, multiple feature combinations are tested. As shown in Table 8, the LBP + RGB combination yields the highest block-level beef detection rate for fat-injected (b_1 − β)%, the highest F1-score (b_F1-Score)%, and the lowest misjudgment rate for non-fat-injected beef (b_α)%. Thus, LBP + RGB is selected as the optimal feature combination.

This section presents small-sample experiments to optimize key parameters of the detection method, including block size, LBP configurations, SVM parameters, and feature combinations. The optimal settings, summarized in Table 9, are then applied to a larger dataset for system performance evaluation.

### 4.2. Performance Results of Large-Sample Experiments

Following small-sample parameter optimization, large-sample experiments were conducted using 600 images across three categories: Wagyu, regular, and fat-injected beef (200 images each). For each category, 120 images were used for training, 40 for validation, and 40 for testing. Each test image was divided into 36 blocks. Details are provided in Table 10.

To compare classification performance, this study evaluates three classifiers: BPN (backpropagation neural network), SVM, and CNN. SVM is chosen for its efficiency and performance with smaller datasets, BPN as a baseline for comparison, and CNN to explore automated feature learning for complex marbling patterns, aligning with state-of-the-art meat quality assessment [9,45]. Optimal SVM parameters from small-sample experiments are used in large-sample testing. Both the CNN and SVM models are evaluated at the block level and aggregated to the image level using majority voting. While the CNN model is trained on segmented image blocks without manual feature extraction, its classification output was interpreted similarly to the SVM approach to ensure comparability across models.

**Figure 17:**
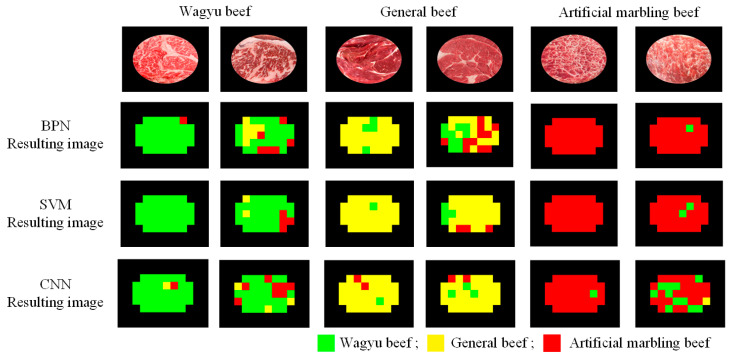
Experimental detection results using different classification models.

Figure 17 shows sample classification results, with performance metrics summarized in Table 11. While CNN outperforms SVM by approximately 5% in both classification accuracy and F1-score at the block and image levels, its longer training time makes SVM the preferred choice for the detection system. Processing times for each classifier are detailed in Table 12.

**Figure 18:**
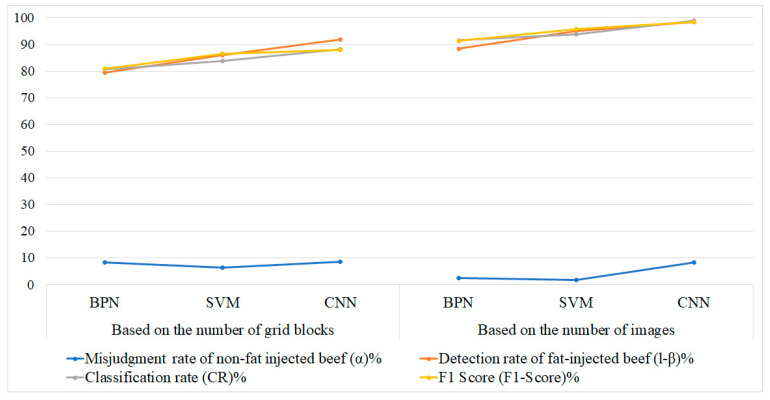
Effectiveness indicators of large-sample experimental results based on block level and image level.

Figure 18 presents effectiveness indicators of large-sample experimental results for detecting artificially marbled beef using BPN, SVM, and CNN models. The left panel shows results based on grid blocks and the right on image counts, including misjudgment rate, detection rate, classification rate (CR), and F1-score. Across both panels, SVM and CNN consistently outperform BPN, with CNN showing slightly higher accuracy, while all models maintain low misjudgment rates. These findings demonstrate the robustness of SVM and CNN in the proposed smartphone-based system, supporting food safety and human-centered healthcare by reliably identifying fat-injected beef.

### 4.3. Impact of External Factors

This study uses a black elliptical mask to define the ROI, divides the image into grid blocks, and extracts features using Uniform LBP and RGB color. To assess the robustness of the proposed fat-injected beef detection method, a sensitivity analysis is conducted using 60 test images (20 per category). The evaluation examines the effects of mask size, image noise (e.g., labels, reflections), brightness changes, and capture angle variations on detection performance.

#### 4.3.1. The Impact of ROI Mask Size on Detection Effectiveness

**Figure 19:**
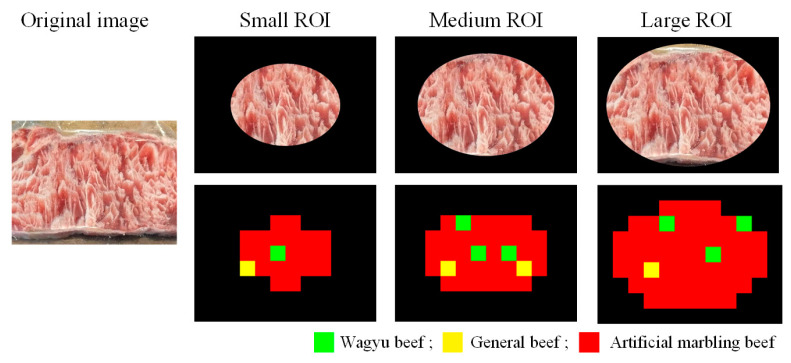
Detection result images with different ROI mask sizes.

**Figure 20:**
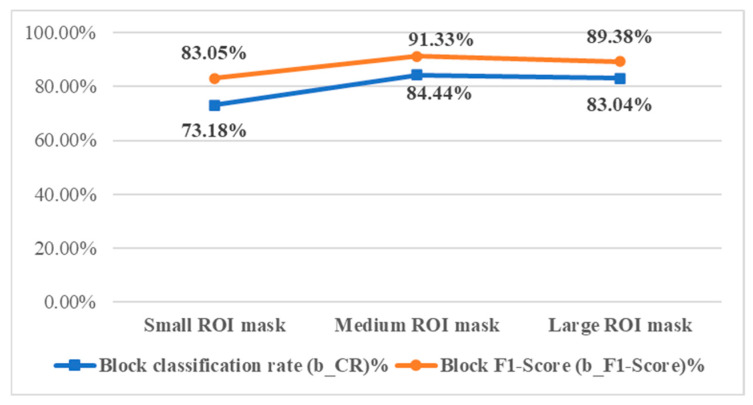
Effectiveness evaluation of detection results with different mask sizes.

The ROI mask in this study ensures that handheld-captured beef images focus on the meat while excluding background noise. Detection performance is evaluated using three mask sizes: smaller masks reduce the detectable area, while larger masks risk including background, which may lower accuracy. As shown in Figure 19 and detailed in Table 13 and Figure 20, the medium-sized mask yields the highest block-level classification rate (b_CR%) and F1-score (b_F1-Score%), making it the optimal ROI mask size for this study.

#### 4.3.2. Impact of Surface Noises on Detection Effectiveness

**Figure 21:**
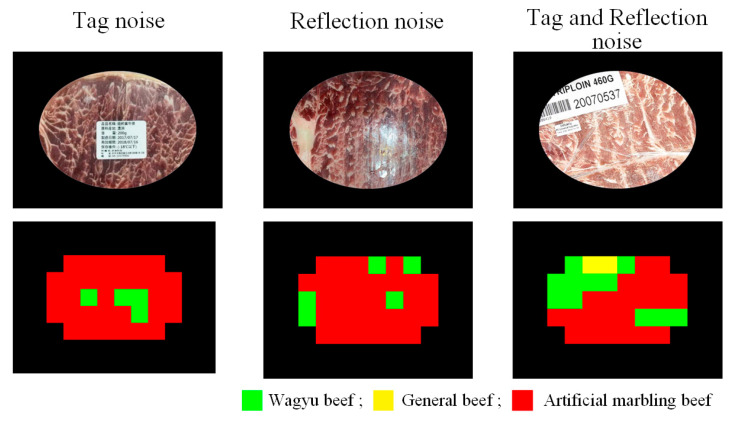
Detection results of images taken under various noise conditions.

**Figure 22:**
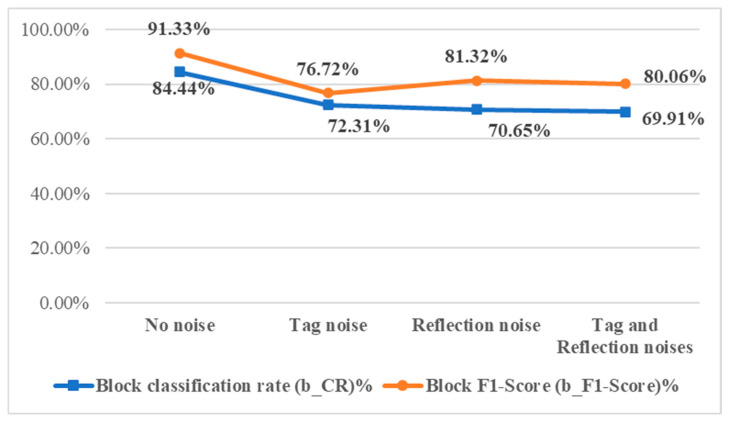
Effectiveness indicators of detection results of testing images with different surface noises.

As the sample images simulate handheld user captures during beef selection, users are advised to avoid label obstruction and minimize reflections from packaging. However, such noise may still occur. This subsection evaluates detection performance on images containing three types of simulated noise: labels, reflections, or both. Figure 21 shows the corresponding experimental results. Figure 22 shows that image noise lowers both the block-level correct classification rate (b_CR%) and F1-score (b_F1-Score%), though accuracy remains above 70%. The highest performance is observed in noise-free images, so users are advised to minimize noise during image capture for optimal results.

#### 4.3.3. Effect of Changes in Image Brightness on Detection Effectiveness

**Figure 23:**
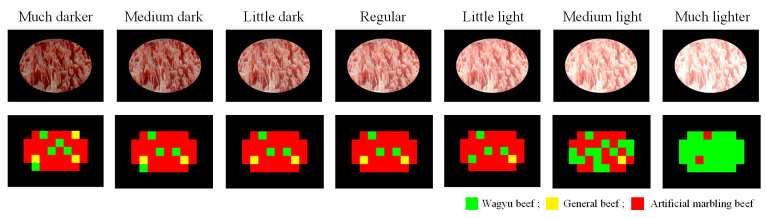
Detection results of images taken under changing ambient brightness.

**Figure 24:**
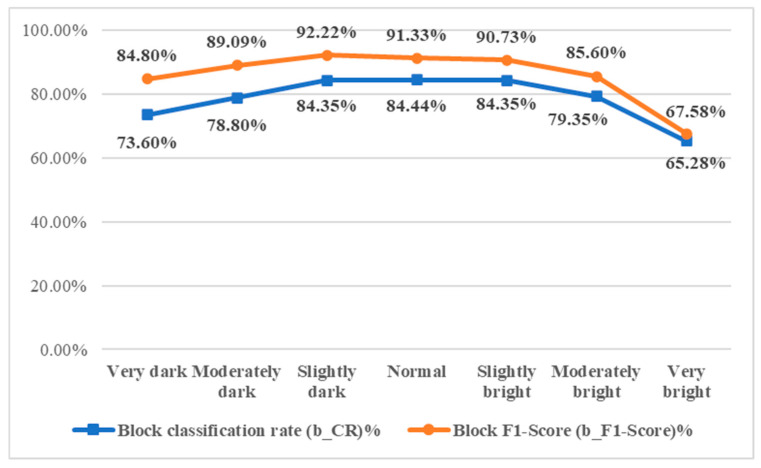
Evaluation of the effectiveness of the detection system under different capture environment brightness.

To assess the effect of brightness on detection performance, this study adjusts the brightness of sample images, which vary due to handheld capture. Mean brightness and standard deviation are calculated, and adjustments are made according to the levels in Table 14. Results are shown in Figure 23, with performance metrics in Figure 24. Normal brightness yields the highest block-level classification rate (b_CR%). While lower brightness slightly reduces performance, both b_CR% and F1-score remain above 70%. High brightness significantly impacts results, with both metrics dropping to around 65%.

#### 4.3.4. Impact of Changing the Image Capture Angle on Detection Effectiveness

**Figure 25:**
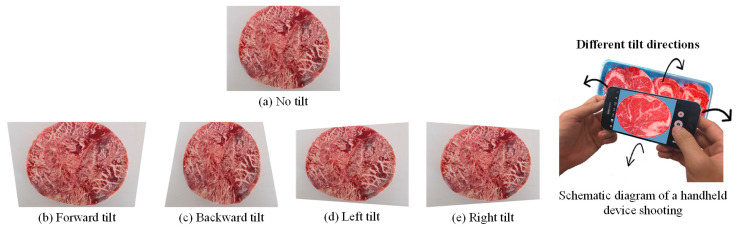
No tilt, forward tilt, backward tilt, left tilt, and right tilt of the camera during image capture.

**Figure 26:**
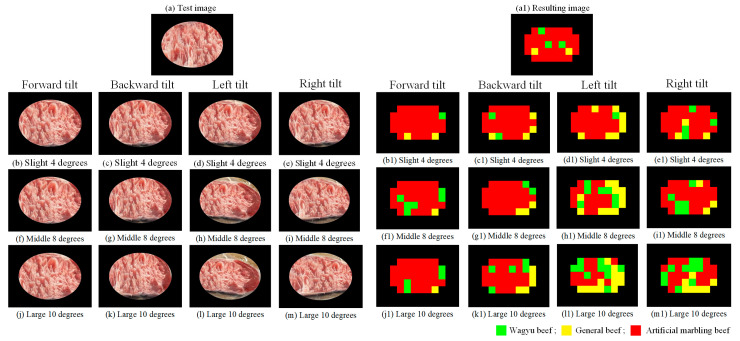
Detection results of images taken at different tilt angles.

**Figure 27:**
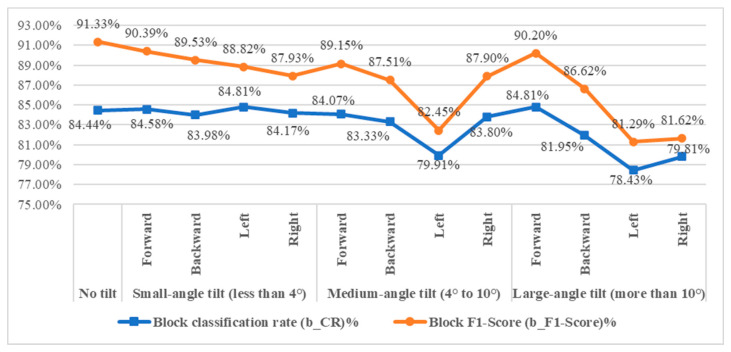
Line chart of the effectiveness of the detection system at various tilt angles.

To simulate real-world handheld imaging, this study examines detection performance under three tilt levels: small (<4°), medium (4–10°), and large (>10°). Tilt refers to angular deviations in the camera’s orientation—up, down, left, or right—common during mobile image capture. The selection of the three tilt levels is based on empirical observations of typical smartphone usage during image capture in retail environments. Consumers often hold their phones at varying angles due to height, lighting, or convenience. These three levels are designed to simulate realistic angular deviations that may occur when users capture beef products on display. Evaluating model performance across these tilt ranges helps to assess the robustness of the system under practical, unconstrained conditions. Sample and result images are shown in Figure 25 and Figure 26. As indicated in Figure 27, increased tilt negatively affects detection performance, with larger angles causing greater declines in block-level classification accuracy.

This section evaluates external factors affecting user-captured images, including ROI mask size, noise, brightness, and capture angles. Results show that small mask sizes slightly reduce block-level classification accuracy (b_CR) but keep it above 80%. Noise from reflections and labels lowers performance, yet b_CR remains around 70%. Brightness variations have a greater effect at higher levels, but b_CR stays above 65%. Tilt angles under 8° have minimal impact, maintaining b_CR above 80%.

### 4.4. Results and Discussion

This study demonstrates the feasibility and effectiveness of a smartphone-based sensing system for identifying artificially marbled beef. The system, using LBP texture and RGB color features with an SVM classifier, achieves an image-level detection rate of 95.00% for fat-injected beef and a misjudgment rate of only 1.67% for non-injected beef. The high F1-score of 95.80% emphasizes the robustness and reliability of the model.

While CNNs outperform SVMs in overall classification accuracy (by approximately 5%), the SVM-based system offers faster processing times and lower hardware requirements, making it more suitable for mobile applications. The robustness analysis confirms that the system maintains acceptable performance even under varied conditions such as brightness changes, surface noise, and image tilt, with classification rates generally above 70%.

Compared to prior methods, such as hyperspectral imaging, chemical analysis, or structured illumination, the proposed approach offers a cost-effective and consumer-accessible solution. Unlike most studies that focus solely on industrial grading, this system empowers consumers to verify meat authenticity in real time.

Limitations include dependency on raw beef images, sensitivity to packaging interference, and performance variations due to environmental factors. Future enhancements should focus on expanding the dataset to include wider beef variations and more diverse samples, improving preprocessing for glare/noise removal, developing a lightweight mobile application with embedded models, and exploring multi-angle and multi-modal image integration. These refinements will further enhance the system’s utility in food fraud prevention and consumer health protection.

Another limitation involves the variability in smartphone camera specifications among consumers. While this study standardized image resolution to 960 × 720 pixels for model input, differences in camera sensors, lenses, and image processing algorithms across devices may affect classification performance. Future studies should include a broader range of smartphone models to evaluate cross-device consistency and further optimize the system for real-world consumer use.

## 5. Concluding Remarks

Ensuring food safety is essential to human-centered healthcare, particularly in addressing risks from fraudulent practices such as beef fat injection. This study contributes to that goal by developing a smartphone-based smart sensing system that empowers consumers to detect artificially marbled beef in real time. The system utilizes LBP texture and RGB color features, classified through an SVM model, offering a practical tool for informed decision making and consumer protection. Large-sample tests reveal that while the CNN slightly surpasses SVM in correct classification rate (CR) and F1-score (differences within 5%), the SVM’s faster processing time makes it the preferred choice for the detection system, achieving a 1.67% misjudgment rate for non-fat-injected beef, a 95.00% detection rate for fat-injected beef, a CR of 93.89%, and an F1-score of 95.80%. By embedding smart sensing technology into purchasing decisions, the system not only alleviates food safety risks but also fosters a proactive healthcare approach by promoting transparency, reducing health disparities, and supporting dietary health management at the point of sale. Future efforts will focus on developing a front-end app and enabling seamless back-end data transmission between the server and the user to fully realize the research objectives.

## Figures and Tables

**Table 1:** Appearance differences of Japanese Wagyu beef and artificially marbled beef.

Name	Wagyu Beef	Artificially Marbled Beef
Captured steak images	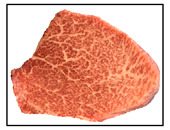	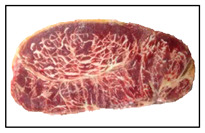
Appearance characteristics	1. Fat appears as dots and streaks, mostly not connected. 2. Fat distribution is scattered and relatively uneven. 3. Larger number of individual fat deposits. 4. Fat thickness varies. 5. Average fat area is relatively small. 6. Fat color varies in intensity; lean meat color is bright red.	1. Fat appears as streaks, most of which are interconnected. 2. Fat distribution shows clear directionality and is relatively uniform. 3. Fewer individual fat deposits. 4. Fat thickness is relatively uniform. 5. Average fat area is relatively large. 6. Fat color is more uniform; lean meat color tends toward dark red.

**Table 2:** Various combinations of texture and color models and corresponding numbers of feature values.

No.	Various Combinations of Texture and Color Models	Number of Feature Values
1	LBP + RGB	59 + 6 = 65
2	LBP + HSV	59 + 6 = 65
3	LBP + CIE L*a*b*	59 + 6 = 65
4	LBP + RGB + HSV	59 + 6 + 6 = 71
5	LBP + RGB + HSV + CIE L*a*b*	59 + 6 + 6 + 6 = 77

**Table 3:** Parameter-setting table of the SVM network in this study.

	Parameter Setting
Input feature vector	65 (Texture features: LBP_0_, LBP_1_, LBP_2_, …, and LBP_58_ (total 59 values) Color features: μ_R_, μ_G_, μ_B_, σ_R_, σ_G_, and σ_B_ (total six values for RGB model)
Penalty parameter (*C*)	Original setting 2^6^ (2^4^, 2^5^, 2^7^, and 2^8^, total five parameters)
Kernel coefficient (*γ*)	Original setting 2^0^ (2^−2^, 2^−1^, 2^1^, and 2^2^, total five coefficients)
Output class	Y_1_ (Wagyu beef), Y_2_ (general beef), and Y_3_ (fat-injected beef)

**Table 4:** Quantity distribution of sample images and grid blocks used in small-sample experiments for parameter setting.

**Small-Sample Experiments**	**Based on Image Level**			
	**Training Images**	**Validation Images**	**Training Images**	**Total**
Wagyu beef	40	20	20	80
General beef	40	20	20	80
Fat-injected beef	40	20	20	80
Total	120	60	60	240
**Small-Sample Experiments**	**Based on Block Level**			
	**Training Images**	**Validation Images**	**Training Images**	**Total**
Wagyu beef	1440	720	720	2880
General beef	1440	720	720	2880
Fat-injected beef	1440	720	720	2880
Total	4320	2160	2160	8640

**Table 5:** Effectiveness indicators using different grid block sizes based on block level and image level (numbers in brackets).

Grid Block Sizes	64 × 64	68 × 68	72 × 72	76 × 76	80 × 80	84 × 84
Misjudgment rates of non-fat-injected beef (b_α)%, (α)%	10.32 (5.00)	8.54 (5.00)	9.94 (5.00)	8.38 (2.50)	7.57 (2.50)	9.55 (5.00)
Detection rates of fat-injected beef (b_l − β)%, (l − β)%	73.82 (85.00)	74.38 (75.00)	71.90 (75.00)	75.68 (80.00)	75.56 (80.00)	78.04 (80.00)
Precisions for fat-injected beef (b_P)%, (P)%	78.15 (89.47)	81.32 (89.47)	78.34 (88.24)	81.87 (94.12)	83.31 (94.12)	80.33 (88.89)
Classification rates (b_CR)%, (CR)%	75.21 (91.67)	77.50 (88.33)	76.07 (88.33)	79.32 (91.67)	79.54 (91.67)	78.63 (90.00)
F1-Scores (b_F1-Score)%, (F1-Score)%	75.29 (87.18)	77.70 (88.28)	74.98 (88.28)	78.65 (86.49)	79.25 (86.49)	79.17 (84.21)

**Table 6:** Comparison table of performance indicators based on block level using different LBP texture feature operators.

LBP_(P, R)_	LBP_(8, 2)_	LBP_(8, 1)_	LBP_(16, 2)_
Schematic diagram of sampling points for different LBP texture features	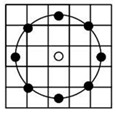	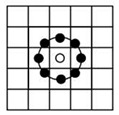	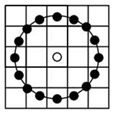
Misjudgment rate of non-fat-injected beef (b_α)%	8.75	7.57	11.53
Detection rate of fat-injected beef (b_l − β)%	74.03	75.56	74.31
Precision for fat-injected beef (b_P)%	80.88	83.31	76.32
Block classification rate (b_CR)%	75.60	79.54	71.30
Block F1-score (b_F1-Score)%	77.30	79.25	75.30

**Table 7:** Classification rates (CR)% based on block level using different parameter combinations of SVM classification model.

	*C* (Penalty Parameter)	16	32	64	128	256
*γ* (Kernel Coefficient)						
0.25		69.40%	72.69%	76.34%	78.56%	78.24%
0.5		72.55%	76.90%	79.07%	78.8%	78.38%
1		76.67%	79.26%	79.54%	78.75%	77.96%
2		79.17%	79.35%	78.56%	77.45%	76.11%
4		78.44%	77.31%	75.37%	73.61%	72.64%

**Table 8:** Comparison of detection effectiveness based on block level using different feature combinations.

Combinations of Feature Types	Misjudgment Rate of Non-Fat-Injected Beef (b_α)%	Detection Rate of Fat-Injected Beef (b_l − β)%	Precision for Fat-Injected Beef (b_P)%	Block Classification Rate (b_CR)%	Block F1-Score (b_F1-Score)%
LBP + RGB	7.57	75.56	83.31	79.54	79.25
LBP + HSV	10.49	73.47	77.79	76.02	75.57
LBP + L*a*b*	10.69	68.89	72.15	74.72	70.48
LBP + RGB + HSV	9.93	74.03	78.85	76.81	76.36
LBP + RGB + HSV + L*a*b*	8.06	73.89	82.10	79.95	77.78

**Table 9:** Preferred parameter settings for the detection method in this study.

Related Parameters	Preference Parameter Selection
Image block size	80 × 80
LBP texture operator configuration	LBP_(8, 1)_
Parameter setting of SVM model	*C* = 64, *γ* = 1
Combinations of feature types	LBP + RGB

**Table 10:** Quantity distribution of sample images and grid blocks used in large-sample experiments for performance evaluation.

**Large-Sample Experiments**	**Based on Image Level**			
	**Training Images**	**Validation Images**	**Testing Images**	**Total**
Wagyu beef	120	40	40	200
General beef	120	40	40	200
Fat-injected beef	120	40	40	200
Total	360	120	120	600
**Large-Sample Experiments**	**Based on Block Level**			
	**Training Images**	**Validation Images**	**Testing Images**	**Total**
Wagyu beef	4320	1440	1440	7200
General beef	4320	1440	1440	7200
Fat-injected beef	4320	1440	1440	7200
Total	12,960	4320	4320	21,600

**Table 11:** Effectiveness indicators of large sample test results of different classifiers.

Classifiers	Effectiveness Indicators	Based on Block Level	Based on Image Level
BPN	Misjudgment rate of non-fat-injected beef (α)%	8.43	2.50
	Detection rate of fat-injected beef (l − β)%	79.31	88.33
	Classification rate (CR)%	80.57	91.67
	F1-score (%)	80.86	91.37
SVM	Misjudgment rate of non-fat-injected beef (α)%	6.48	1.67
	Detection rate of fat-injected beef (l − β)%	85.93	95.00
	Classification rate (CR)%	83.81	93.89
	F1-score (%)	86.41	95.80
CNN	Misjudgment rate of non-fat-injected beef (α)%	8.52	8.33
	Detection rate of fat-injected beef (l − β)%	91.94	98.33
	Classification rate (CR)%	88.07	98.89
	F1-score (%)	87.99	98.33

**Table 12:** Efficiency indicators of different classifiers for large sample images in this study.

Processing Time of Classifiers	BPN	SVM	CNN
Training time (Min.)	8.09 min	3.83 min	38.82 min
Testing time (S/image)	0.12 s	0.08 s	0.16 s

**Table 13:** Specification comparison table of different ROI mask sizes.

ROI Mask Types	Small ROI Mask	Medium ROI Mask	Large ROI Mask
Mask specifications	Long axis: 576 pixels Short axis: 432 pixels	Long axis: 720 pixels Short axis: 540 pixels	Long axis: 864 pixels Short axis: 648 pixels
Area of small ROI mask: 196,145 pixels Area of medium ROI mask: 302,783 pixels Area of large ROI mask: 440,813 pixels	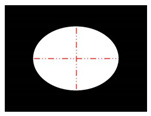	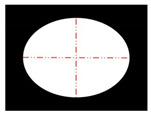	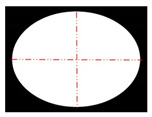
Example images	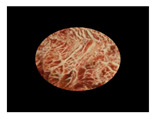	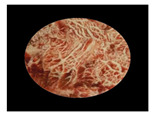	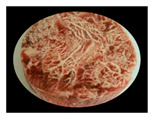

**Table 14:** Names and intervals of different brightness levels in image capture environments.

Brightness Levels	Very Dark	Moderately Dark	Slightly Dark	Normal	Slightly Bright	Moderately Bright	Very Bright
Brightness standard	μ − 4.5σ	μ − 3σ	μ − 1.5σ	μ	μ + 1.5σ	μ + 3σ	μ + 4.5σ
Average brightness	21.01	46.98	72.94	98.91	124.88	150.85	176.82

## Data Availability

The data will be made available on request.
